# Tracking and Treating Malignant Melanoma Metastases

**DOI:** 10.1155/2012/173491

**Published:** 2012-03-07

**Authors:** Gérald E. Piérard, Philippe Humbert, Pascale Quatresooz

**Affiliations:** ^1^Department of Dermatopathology, University Hospital of Liege, 4000 Liege, Belgium; ^2^Department of Dermatology, University Hospital, 25000 Besançon, France; ^3^University of Franche-Comté, 25000 Besançon, France; ^4^Inserm U645 Research Unit IFR133, Besançon, France

Cutaneous malignant melanoma (CMM) is a neoplasm which has always evoked scientific interest out of proportion to its frequency. Over the past decades, CMM has shown the most rapidly increasing neoplasm in yearly incidence. Such increased incidence outweighed the moderate improvements in therapy, causing a global increase in mortality. The disease progresses from the primary CMM (stages I and II) to a locoregional disease (stage III) and a disseminated metastatic disease (stage IV). Surgery represents the mainstay often curative for thin primary CMM that have not yet given rise to distant metastases. Beyond that stage, the therapeutic options remain unsatisfactory. Indeed, according to the American Joint Committee on Cancer (AJCC), the percentage of patients with metastatic melanoma surviving 1-year ranges from 33 to 62%.

The present special issue contains ten papers focused on new developments in the field of CMM progression toward the metastatic disease. Two papers deal with imaging procedures used for detecting metastases and with unusual clinical evolution of metastases. Two papers focus on serum biomarkers, and one covers the interpretation of CMM cell migration along specific skin structures. A series of four papers regard peculiar aspects of molecular biology associated with the CMM metastatic process. Finally, one paper addresses some recent developments in biologic treatments.

In the paper entitled “*Review of diagnostic imaging modalities for the surveillance of melanoma patients,*” Y. Xing et al. present a meta-analysis evaluating the current state of imaging modalities for detecting CMM metastases. They compare the sensitivity, specificity, and positive predictive values of ultrasonography, computed tomography (CT), positron emission tomography (PET), and CT-PET combined. Ultrasonography was found to be the most sensitive and specific for detecting lymph node metastases, and PET-CT was the most sensitive and specific for detecting distant metastases.

In the paper entitled “*Smouldering malignant melanoma and metastatic dormancy*,” G. E. Piérard et al. explore two peculiar evolutions of the versatile CMM metastatic disease, namely, the smouldering CMM and the CMM dormancy. A long disease-free interval (CMM dormancy) occasionally occurs before the surge of overt metastases. The so-called CMM smouldering phenomenon refers to the condition where regional metastases wax and wane for long periods of time on restricted skin regions. Local micrometastases often predict sentinel lymph node involvement but they do not always reflect progression of the primary CMM to full-blown visceral metastatic competence. A combination of factors impacts the versatile CMM metastasic progression. The putative factors include the heterogeneity and phenotypic variability of CMM cells, the presence of CMM stem cells and CMM cells engaged in an amplification proliferation pool, as well as the host immune response, and possibly the induction of a particular stromal structure and vascularity.

The papers entitled “*Biomarkers as key contributors in treating malignant melanoma metastases*,” by C. Ferreira de Souza et al., and “*A synopsis of serum biomarkers in cutaneous melanoma patients,*” by P. Vereecken et al., describe CMM serum biomarkers. A poor correlation exists between biomarkers discovered by basic research and data from clinical trials. It remains that LDH and the S100 B protein levels are correlated with poor prognosis in the American Joint Committee of Cancer (AJCC) stage III/IV CMM patients.

The paper entitled “*Thigmotropism of malignant melanoma cells,*” by P. Quatresooz et al., is an original view of the migration process of CMM cells. During CMM progression including incipient metastasis, neoplastic cells follow some specific migration paths inside the skin. In particular, they progress along the dermoepidermal basement membrane, the hair follicles, the sweat glands, nerves, and the near perivascular space. These features evoke the thigmotropism phenomenon defined as a contact-sensing growth of cells. This process is likely connected to modulation in cell tensegrity (control of the cell shape). These specifically located paucicellular aggregates of CMM cells do not appear to be involved in the tumorigenic growth phase, but rather they participate in the so-called “accretive” growth model.

In the paper entitled “*Molecular dermatopathology in malignant melanoma,*” M. A. Reginster et al. summarize and update some methods of molecular diagnosis applicable to CMM. Sensitive techniques are available for detecting specific DNA and RNA sequences by molecular hybridization. Cytogenetics highlights the pathogenesis of atypical melanocytic neoplasms with emphasis on the activation of the mitogen-activated protein kinase (MAPK) signalling pathway during the initiation step of the neoplasms. A minority of CMM families have mutations in the tumour suppressor gene p16 or CDKN2A. In addition, somatic mutations in p16, p53, BRAF, and cKIT are present in CMM. Genome-wide scan analyses on CMM indicate positive associations for genes involved in melanocytic naevi, but MM is likely caused by a variety of common low penetrance genes.

The paper entitled “*miRNAs and melanoma: how are they connected?*” by A. Taveira da Cruz et al. is focused on the epigenetic and mRNA control by microRNAs (miRNAs). miRNAs are essential in the processing and control of many biological responses, in particular in cancer development. Some molecular pathways are frequently disrupted in CMM, and miRNAs probably have a decisive role on that. New findings about miRNAs are presented in CMM, underlying both some epigenetic processes, and the promise of miRNAs in diagnosis and therapy of CMM.

In the paper entitled “*RAS/RAF/MEK/ERK and P13K/PTEN/AKT signalling in malignant melanoma progression and therapy,*” I. Yajima et al. discuss molecular changes associated with CMM progression. In human CMM, the RAS/RAF/MEK/ERK (MAPK) and the P13K/PTEN/AKT (AKT) signalling pathways represent two major pathways that are constitutively activated through genetic alterations. Mutations of RAF, RAS, and PTEN contribute to anti-apoptosis, abnormal proliferation, angiogenesis, and invasion for CMM development and progression. This paper reviews the MAPK and AKT signalling networks associated with CMM development and progression.

In the paper entitled “*Targeting the cellular signalling: BRAF inhibition and beyond for the treatment of metastatic malignant melanoma,*” F. Ades and O. Metzger-Filho report important translational researches having identified mechanisms in malignant transformation, invasion, and progression. Signalling pathways can be abnormally activated by oncogenes. The identification of oncogenic mutated kinases in CMM provides an opportunity for new target therapies. The CMM dependence of BRAF-mutated kinase allowed the development of inhibitors that produced major responses in clinical trials. This represents the beginning of a novel class of drugs in metastatic CMM; the identification of the transduction signalling networking and other “druggable” kinases is currently under active investigations. In this review, the ongoing research on cellular signalling inhibition, resistance mechanisms, and strategies to overcome treatment failure is discussed in depth.

In the paper entitled “*Ipilimumab, a promising immunotherapy with increased overall survival in metastatic melanoma?*”, G. E. Piérard et al. review new advances in metastatic CMM biotreatment. The therapeutic options remain limited for advanced CMM, and those directed to the neoplastic cells have not brought major survival advantage so far. Immunotherapy is another targeted option. Ipilimumab, a monoclonal antibody directed to CTLA-4 present on cytotoxic T cells, boosts immunity, particularly its anti-CMM activity. Under treatment, the overall survival of patients with CMM metastases is moderately but significantly increased. In some instances, the immunorelated adverse effects are severe and life-threatening.

We hope this special issue will not only help to clarify the understanding of the CMM metastatic process, but also will reinforce the development of better treatment modalities for disseminated CMM

**Gérald E. Piérard** **Gérald E. Piérard** **Philippe Humbert** **Philippe Humbert** **Pascale Quatresooz** **Pascale Quatresooz**

